# Toward Smart Aerospace Structures: Design of a Piezoelectric Sensor and Its Analog Interface for Flaw Detection

**DOI:** 10.3390/s141120543

**Published:** 2014-10-31

**Authors:** Hamza Boukabache, Christophe Escriba, Jean-Yves Fourniols

**Affiliations:** 1 ECA Group, Aerospace Division, 5 Rue Paul Mesple, Toulouse 31100, France; 2 CNRS, LAAS, 7 Avenue du Colonel Roche, Toulouse F-31400, France; E-Mails: cescriba@laas.fr (C.E.); fourniols@laas.fr (J.-Y.F.); 3 Univ de Toulouse, INSA, LAAS, Toulouse F-31400, France

**Keywords:** structural health monitoring (SHM), smart piezoelectric sensors, aircraft composite structures, guided waves, Lamb waves, sensor analog interface, avionics

## Abstract

Structural health monitoring using noninvasive methods is one of the major challenges that aerospace manufacturers face in this decade. Our work in this field focuses on the development and the system integration of millimetric piezoelectric sensors/ actuators to generate and measure specific guided waves. The aim of the application is to detect mechanical flaws on complex composite and alloy structures to quantify efficiently the global structures' reliability. The study begins by a physical and analytical analysis of a piezoelectric patch. To preserve the structure's integrity, the transducers are directly pasted onto the surface which leads to a critical issue concerning the interfacing layer. In order to improve the reliability and mitigate the influence of the interfacing layer, the global equations of piezoelectricity are coupled with a load transfer model. Thus we can determine precisely the shear strain developed on the surface of the structure. To exploit the generated signal, a high precision analog charge amplifier coupled to a double T notch filter were designed and scaled. Finally, a novel joined time-frequency analysis based on a wavelet decomposition algorithm is used to extract relevant structures signatures. Finally, this paper provides examples of application on aircraft structure specimens and the feasibility of the system is thus demonstrated.

## 1. Introduction

Over the past twenty years, the aerospace industry has registered a high mutation and evolution due to the use of new composite materials. This constant integration has led to the development of new hybrid aircrafts, lighter and boasting more autonomy than the previous generation. The growing complexity of aircraft structures makes maintenance more difficult and time consuming, especially for composite parts. The intrinsic nature of carbon fiber stratified materials makes the structures very sensitive to external shocks which are the main factor in the appearance of delamination [1,2]. Therefore hidden local flows can appear throughout the thickness of the structure without any visual exterior sign and weaken their global mechanical proprieties. As a direct consequence, commercial flying companies began using, aside from the traditional visual inspection, new commercial nondestructive evaluation tools. However, typically based on non-intrusive local methods, these tools are unsuitable for the inspection of very large structures. They are generally based on the use of compression ultrasonic waves to probe the structure thickness [3] or eddy currents [4] to probe the structure surface. However, to cover large areas, these very local techniques need extensive human intervention and a long immobilization of the planes, which is costly and time consuming.

**Figure 1.**
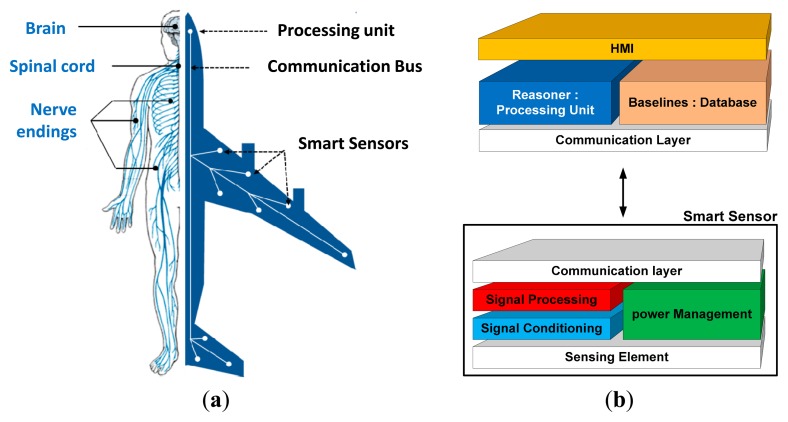
(**a**) Diagnostic system: Analogy with the human body (**b**) Layer model of the proposed smart sensor.

To make the inspection more autonomous, we developed a new methodology based on a smart sensor capable of covering large areas made from different kind of materials. Based on the reversible capability of piezoelectric materials to generate and sense periodical strain, our principle exploits the generation of guided wave to detect flaws. Inspired by the human body and its nervous system (Figure 1a), the proposed final system is based on a smart distribution of multiple communicating piezoelectric-based smart sensors (Figure 1b). On the other hand, a central processing unit is in charge of data collection and diagnosis calculation. The sensor network development begins by the node design and therefore by the piezoelectric sensor scaling. Thereby, in this paper we present an analytical study of the sensor/actuator development and correlate the developed model to the experimental results. We present how we can generate and sense Lamb waves and the way to exploit them to identify mechanical damages. Finally, the sensor integration is presented and feasibility experiments are shown for different aircraft part specimens.

## 2. Methodology

**Figure 2.**
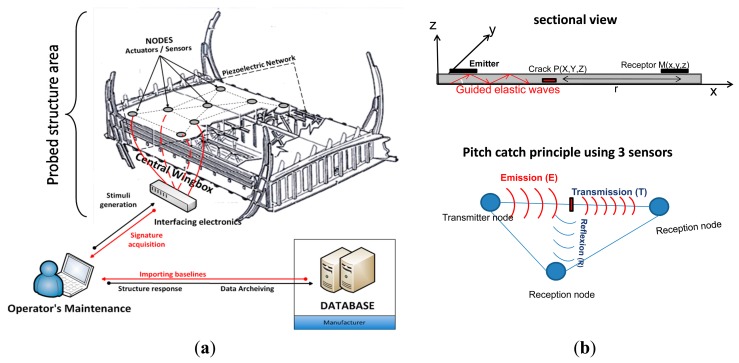
(**a**) Synoptic of the damage diagnosis system; (**b**) Pitch catch technique: one of the used method to extract structures acoustic signature.

To make the regular nondestructive inspections time efficient and thus reduce the global cost linked to the maintenance procedures, we have developed a sensor network based on piezoelectric patches that would be capable to detect on the ground, delaminations in carbon fiber reinforced polymer as well as disbonds. Based on the capability of PZT actuators/sensors to generate/sense guided waves into the host structure, we exploit theses waves to detect any eventual damage using the pitch-catch technique (Figure 2b). The diagnosis method is based on a comparison between the global acquired signals and a baseline registered before the commissioning of the plane (Figure 2a). A maintenance operator uses therefore a database where each part's signature is registered and compares it to the new structure response. The identification of damage is performed using multiple algorithms for time-frequency analysis based on wavelet transforms.

## 3. Sensor/Actuator Development

**Figure 3.**
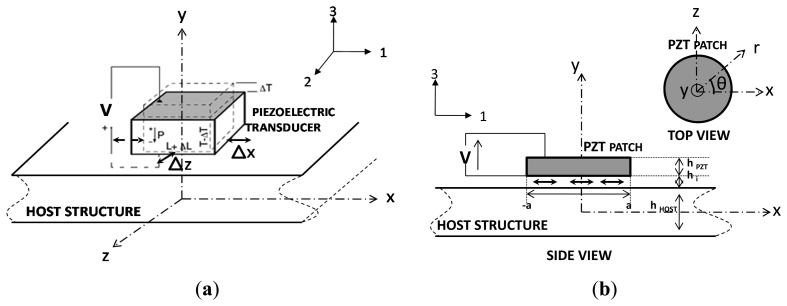
(**a**) Piezoelectric transducer mounted onto a host structure surface; (**b**) Circular piezolectric patch pasted onto a host structure using an adhesive layer.

Lamb waves in structures can be generated with more less efficiency using different techniques such as wedge coupled angle ultrasonic probes [5], comb ultrasonic transducers [6] or inter-digited electrode array transducers [7]. Although preferred for their high precision, these techniques are however very local and quite difficult to scale. Therefore, the focus of our research turned to the study of small transducers made of piezoelectric material mounted directly onto the surface of the structure without intrusion (Figure 3a). The exploitation of the piezoelectric effect allows for the generation of a shear stress through the interfacing layer onto the structure (Figure 3b). This transmitted stress is at the origin of Lamb wave generation [8]. Thereby, based on the duality of the piezoelectric material, we are capable using a single transducer to generate and measure theses waves and finally exploit them for damage detection.

### 3.1. Piezoelectric Transducer Scaling and Characterization

According to the piezoelectric effect (Equation (1)), the application of a bipolar sinusoidal voltage on the transducer develops an alternative material displacement following the three axes 1, 2 and 3. The phenomenon is totally reversible. Thus, the sensor generates an electric field when it is subjected to a mechanical stress [8]:

(1) $$\left\{\begin{array}{c} Q_{1}\\ Q_{2}\\ Q_{3}\\ S_{11}\\ S_{22}\\ S_{33}\\ S_{23}\\ S_{13}\\ S_{12} \end{array}\right\}=\left[\begin{array}{ccccccccc} P_{1} & 0 & 0 & 0 & 0 & 0 & 0 & d_{15} & 0\\ 0 & p_{2} & 0 & 0 & 0 & 0 & d_{15} & 0 & 0\\ 0 & 0 & p_{3} & d_{31} & d_{31} & d_{33} & 0 & 0 & 0\\ 0 & 0 & d_{31} & c_{11} & c_{12} & c_{33} & 0 & 0 & 0\\ 0 & 0 & d_{31} & c_{12} & c_{11} & c_{13} & 0 & 0 & 0\\ 0 & 0 & d_{33} & c_{13} & c_{13} & c_{33} & 0 & 0 & 0\\ 0 & d_{15} & 0 & 0 & 0 & 0 & c_{55} & 0 & 0\\ d_{15} & 0 & 0 & 0 & 0 & 0 & 0 & c_{55} & 0\\ 0 & 0 & 0 & 0 & 0 & 0 & 0 & 0 & c_{66} \end{array}\right]\left\{\begin{array}{c} V_{1}\\ V_{2}\\ V_{3}\\ T_{11}\\ T_{22}\\ T_{33}\\ T_{23}\\ T_{13}\\ T_{12} \end{array}\right\}$$

where Q*_i_* and V*_i_* are respectively the charge generation (Coulomb) and the voltage vector (Volts). T*_ii_* and S*_ii_* are the stress and strain vectors respectively. *d, p* and *c* denote the piezoelectric strain constant matrix, dielectric permittivity and finally the compliance constant matrix.

The material's characteristics determine the transducer's behavior and thus the excitation which is transmitted to the structure. Therefore, a comparison study between different piezoelectric materials was performed to find out the best candidate for sensor integration (Table 1a). Because of its excellent electromechanical efficiency k_31_ = 30%, we focused our research on Lead Zirconite Titanate (PZT). Finally, among the PZT family we chose to use PZT-5A (Table 1b). This material showed a high piezoelectric charge/force ratio and good voltage constants, respectively d_31_ = −175 × 10^−12^·C·N^−1^ and g_31_ = 12.4 × 10^−3^·Vm·N^−1^ which insure a high electromechanical coupling. Electrode design and geometry of the PZT transducers are also critical because they directly affect the sensor's operating point. They fix the sensor vibration orientation and set the operating oscillation frequency. According to Equation (1), the relation between the electrical field and the mechanical strain is defined as:

(2) $$\left\{\begin{array}{c} S_{1}\\ S_{2}\\ S_{3}\\ S_{23}\\ S_{13}\\ S_{12} \end{array}\right\}=\left[\begin{array}{ccc} 0 & 0 & d_{31}\\ 0 & 0 & d_{32}\\ 0 & 0 & d_{33}\\ 0 & 0 & 0\\ d_{15} & 0 & 0\\ 0 & 0 & 0 \end{array}\right]\left\{\begin{array}{c} E_{1}\\ E_{2}\\ E_{3} \end{array}\right\}$$

where *E* is the electric field (V/m) vector. With electrodes on the top and the bottom of the transducer as shown in Figure 3a, the created electric field is normal and therefore the strains and the displacements are in all directions:

(3) $$\{\begin{array}{c} S_{1}=d_{31}E_{3}\\ S_{2}=d_{32}E_{3}\\ S_{3}=d_{33}E_{3} \end{array}$$

**Figure 4.**
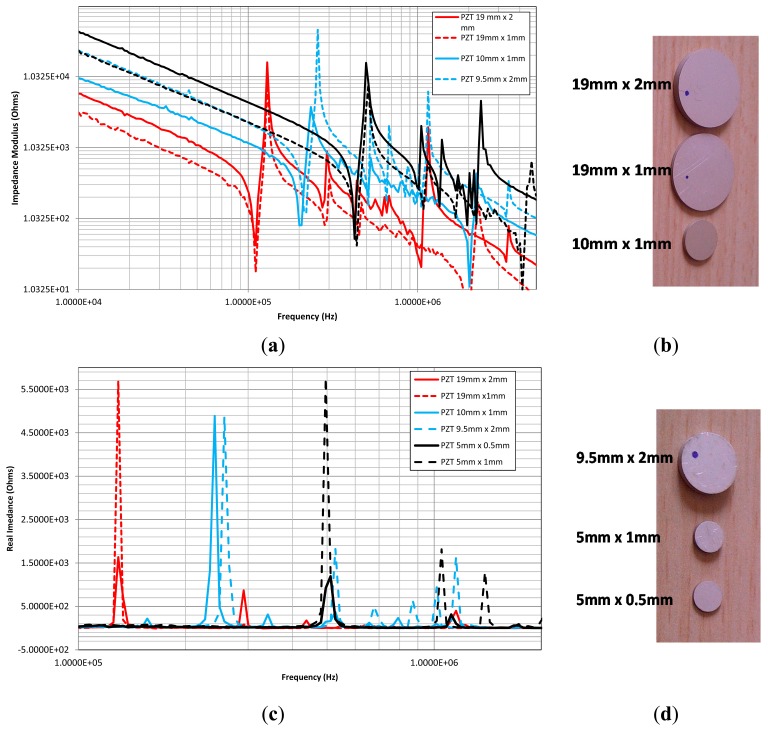
(**a**) Impedance modulus of the sensors between 100 kHz and 1.2 MHz. The study was performed using an Agilent 4294 impedance analyzer; (**b**) picture of different PZT sensor sizes: 19 mm of diameter and 2 mm thick, 19 mm of diameter and 1 mm thick, 10 mm of diameter and 1 mm thick, respectively; (**c**) Real part impedance of the different sensors, the spikes identify the anti-resonance frequency; (**d**) Picture of the remaining PZT sensors and their size.

In order to have a radial divergent strain we designed the transducer to have a disc shape. This insures a uniform distribution of the generated displacement onto the structure all around the sensor (Figure 3b). The discs have different diameters (5–19 mm) and different thickness (2–0.5 mm). These two parameters fix the radial vibration frequency in the range (100–400 kHz) and the normal vibrations in the range (1–4 MHz). Thus, we obtained a good decoupling between the two vibrating modes (Figure 4).

### 3.2. Piezoelectric Transducer Interfacing with Host Structure

Providing perpendicular polarisation V to the piezoelectric circular patch (*cf*. Figure 3b) simplifies Equation (1) and creates a radial and angular strain respectively *S_r_* and *S*_θ_ which are more readily expressed in polar coordinates:

(4) $$s_{r-\text{PZT}}=s_{r-\theta }=d_{31}\frac{V}{h_{PZT}}$$

The transmission of this created actuation to the structure is performed through a bonding layer that acts as a shear layer. Considering the thin size of this interface the strain distribution can be assumed linear throughout its thickness. Therefor by using Carwley 1D model [9–11] describing the transmission of a mechanical actuation through an elastic layer we computed the transmitted strain *s_PZT-1_* and *s_Host-1_* at the interface between the PZT patch and the host structure (Figure 3b). The calculation of the actuation displacement *u_PZT-1_* and the host surface structure displacement *u_Host-1_* is thus performed by integrating the strain equations:

(5) $$S_{\text{PZT}-I}\left(x\right)=\frac{\alpha }{\alpha +\psi }S_{r-PZT}\left(1+\frac{\psi }{a}\frac{\cosh\left(\Gamma \text{os}\right)}{\cosh\left(\Gamma a\right)}\right)$$

(6) $$S_{\text{HOST}-I}\left(x\right)=\frac{\alpha }{\alpha +\psi }S_{r-PZT}\left(1-\frac{\cosh\left(\Gamma \text{os}\right)}{\cosh\left(\Gamma a\right)}\right)$$

with:

$$\psi =\frac{Et}{E_{a}t_{a}}\;\text{and}\;\Gamma^{2}=\frac{G_{b}}{E_{a}}\frac{1}{t_{a}t_{b}}\frac{\alpha +\psi }{\psi }$$

**Figure 5.**
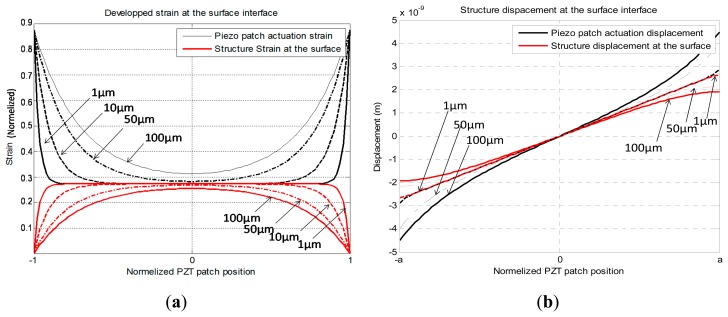
(**a**) Developed strain calculed in the interface area between the transducer and the host structure; (**b**) Developed displacement at the interface area between the transducer and the host structure. Notice that for a bonding interface thikness equal to 1 μm, the transducer displacement actuation is equal to the induced host surface displacement.

**Figure 6.**
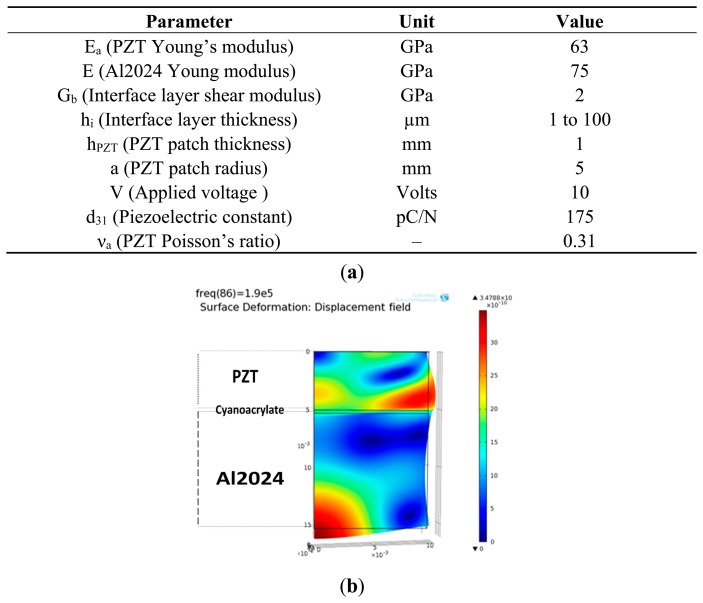
(**a**) Simulation parameters; (**b**) FEM simulation of the global displacement field that PZT patch produces on the host structure. The simulation was performed using parameters and for an epoxy bonding of 1 μm thickness.

Using Equations (5) and (6) and the simulation parameters presented in Figure 6a, we computed the developed strain and the generated displacement. The results are presented in Figures 5 and 6b. From Figures 5 and 6a, we deduced that, for an ideal bonding, the interface layer should tend to zero or have an infinite young modulus. The calculations show that a thin adhesive layer (less than 1 μm) produces a good shear mechanical displacement transfer between the transducer and the structure.

In this scenario, almost all the mechanical load is concentrated at the transducer's edges. When the bounding layer thickness *h_i_* tends to zero, the produced shear stress is only concentrated at the infinitesimal edge of the transducer. Therefore we can represent it as a function of Dirac operator δ(*x*):

(7) $$T_{\text{Interface}}=T_{0}\;[\delta (x-a)-\delta (x+a)]$$

Therefore, to insure a good mechanical match between the sensors and the structures, we used cyanoacrylate glue and epoxy with a shear modulus G_b_ of 0.7 GPa and 2 GPa, respectively.

### 3.3. Piezoelectric Signal Conditioning

According to the piezoelectric equation (Equation (1)), the designed piezoelectric patches (Figure 1), when subjected to a mechanical force or stress, act as a charge generators. Equation (1) can be simplified in the polar coordinates system, referring to Figure 3, as:

(8) $$D_{3}=d_{31}(T_{r}+T_{\theta })+\varepsilon_{33}^{T}\;E_{3}$$

Compared to the structure, the piezoelectric patch is relatively small. The sensed tangential strain and radial strain can therefore be assumed equals and are noted *S*. Hence, in the absence of external polarization, the generated electric charges displacements equation is given by:

(9) $$D_{3}=d_{31}(T_{r}+T_{\theta })=\frac{d_{31}E_{a}}{1-\upsilon_{a}}\cdot 2S$$

The total charge *Q* generated by the sensor on the top and the bottom surface electrodes is equal and is calculated by integration of the electric displacement over the sensor area [12], upon applying Gauss' theorem:

(10) $$Q=\frac{d_{31}E_{a}}{4\pi \cdot (1-\upsilon_{a})}\cdot \iint 2S\cdot r\cdot dr\cdot d\theta$$

Substituting the strain by the deformation Δ*u_a_* formula and upon simplification we finally find the expression of the sensor's charge generation:

(11) $$Q=\frac{a\cdot d_{31}E_{a}}{4\cdot (1-\upsilon_{a})}\cdot \Delta u_{a}$$

**Figure 7.**
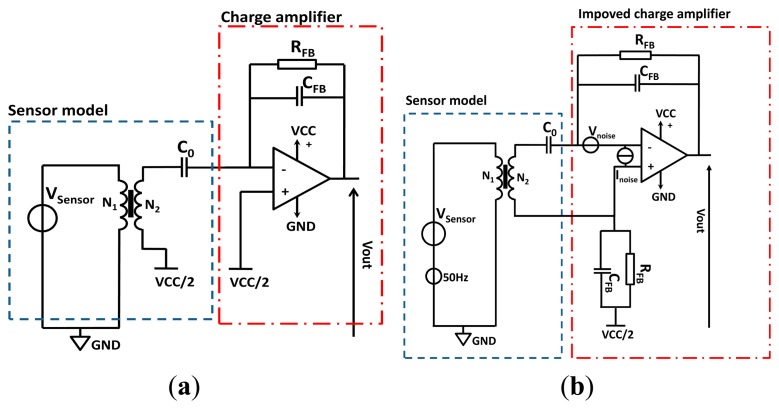
(**a**) PZT Sensor and bounding model with a conditioning circuit; (**b**) PZT Sensor and bonding model with an improved conditioning circuit using differential inputs. Notice that the model includes the parasitic signals inputs.

Therefore, when the piezoelectric sensor is in passive mode, we model its temporal behaviour by a current source in parallel with the sensor parasitic capacitance C_0_. Using the Thevenin/Norton [13] duality theorem, the sensor can be modelled by a voltage source in series with C_0_. On the other hand, the effect of the pasting is modelled by a passive transformer as shown in Figure 7. The ratio between N_1_ and N_2_ of the transformer is a simple image of the degradation that the interfacing layer induces to the charges generation. Hence, when the bonding is ideal, N_1_ is equal to N_2_.

To exploit the delivered signal, a classical charge amplifier is used for signal conditioning. It converts a charge displacement to an exploitable voltage with a minimal loss and performs the impedance matching between the sensor and the rest of the electronic circuit [14]. The amplifier circuit presents to the piezoelectric sensor an input that is virtually a ground which allows us to collect most of the generated charge. The high frequency gain of the charge amplifier is fixed by C_FB_:

(12) $$\text{Gain}=\frac{1}{C_{FB}}(mV/C)$$

To insure the biasing of the amplifier, a feedback resistance R_FB_ is needed to provide the needed DC current bias at the input of the OPA. However, this resistance affects the bandwidth of the circuit which then behaves as a high-pass filter, presenting a cut off frequency at:

(13) $$f_{3dB}=\frac{1}{2\pi R_{FB}C_{FB}}$$

To size the gain of the charge amplifier and determine the values of R_FB_ and C_FB_, we need to have an idea about the quantity of charges that the piezoelectric sensor can generate. To do that we need to know the value of the deformation that will affect the sensor.

**Figure 8.**
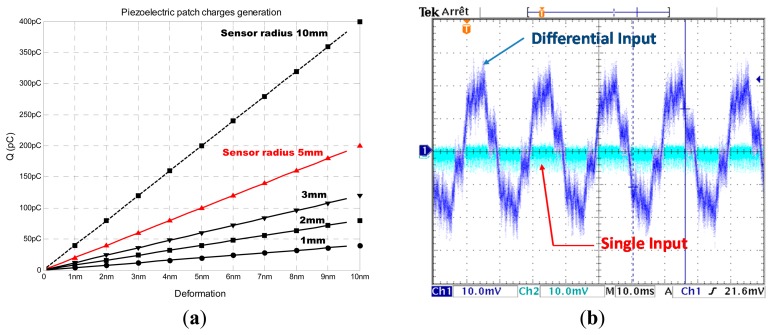
Piezoelectric charge conversion to exploitable voltage. (**a**) Piezoelectric charges generation for differents radial deformations and differents sensor sizes; (**b**) Noise performance of a differential input charge amplifier versus a single input circuit.

To simplify the calculation of the generated charge, we may assume that there is no energy loss into the host structure we can make the simple hypothesis that the generated deformation produced by a PZT actuator is equal to the deformation sensed by the same PZT in passive mode. Based on this assumption, on Equation (11) and on the simulation parameters shown in the table of Figure 6a we quantified as presented in Figure 8 the charge generation. If we fix an operating point at 100 pC and we need to have a voltage output given by the charge amplifier equal to 1 V, the relation (12) yields a C_FB_ equal to 100 pF.

However, due to the high gain, the charge amplifier presented in Figure 7a is a very sensitive circuit. Any capacitive coupling introduced by the sensor with the input, in our case due to the 50 Hz power (Figure 8b), will inject current. In the case of the circuit shown in Figure 7a, the injected parasitic current is amplified which make the measurement of piezoelectric injected charges quite complex. In the case of the improved circuit shown in Figure 7b, the common mode signals applied on the differential inputs of the amplifier cancel each other (Figure 8b).

**Figure 9.**
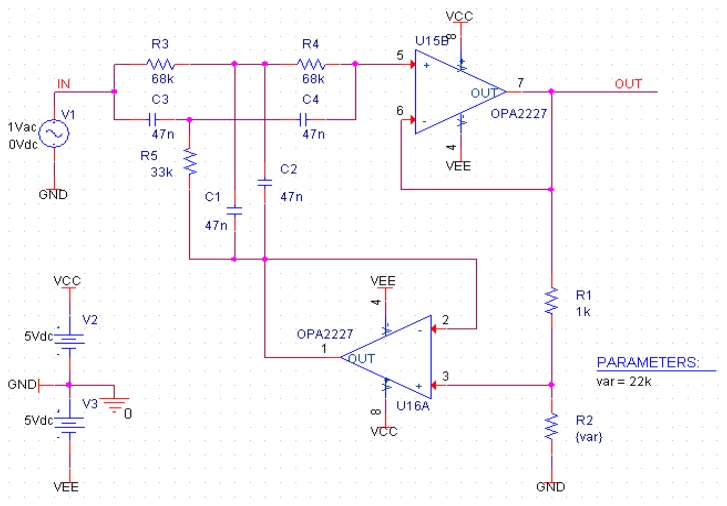
Schematics of double T 50 Hz notch filter topology.

To improve the signal-to-noise ratio, we added a double T 50 Hz notch filter (Figure 9) at the output of the charge amplifier. It allowed us to remove the parasitic signal due to power lines and plugs.

**Figure 10.**
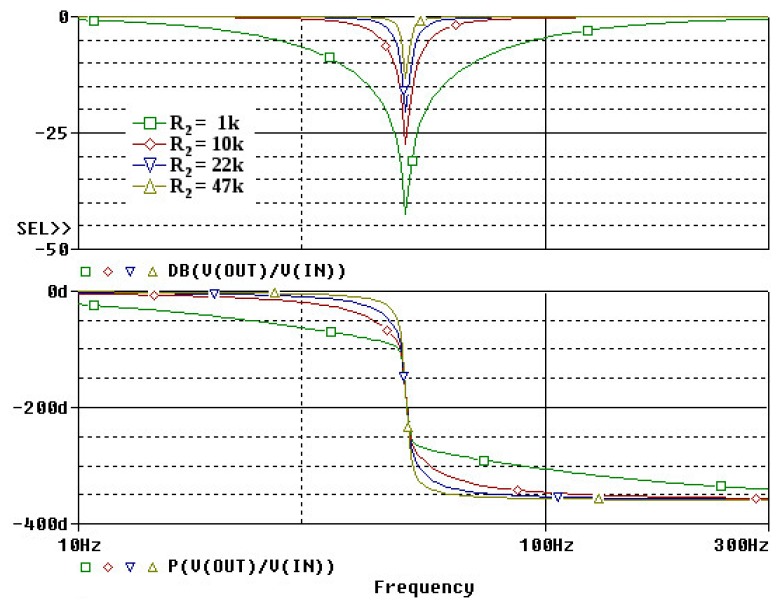
Bode response of the used double T 50 Hz notch filter.

**Figure 11.**
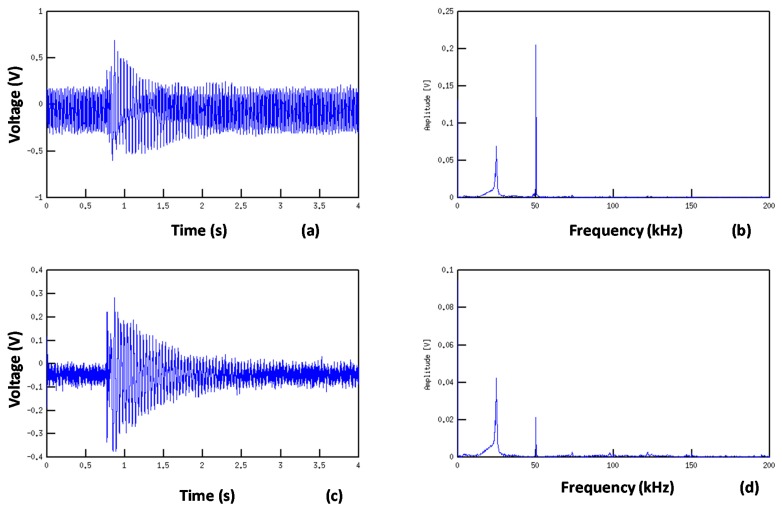
Temporal filter response: (**a**) Non filtred temporal signal; (**b**) Spectral response of the signal. Notice the amplitude of the 50 Hz; (**c**) Filtred temporal signal using the 50 Hz notch filter; (**d**) Spectral response of the filtered signal. Notice the reduction of the 50 Hz noise.

This notch filter topology provides good results despite the variations of the values of the passive components (Figure 10). The filter's cut-band and attenuation is fixed by the ratio of R1/R2. For lower ratios, the filter has less attenuation but a shorter cut-band. For this application, we chose R2 = 22 k which provides an attenuation of about 20 dB for a cut-band of 10 Hz around the 50 Hz noise (Figure 10). Thus we don't attenuate the modal frequencies nearby (Figure 11). One important side-effect of using this filter is that by eliminating some of the 50 Hz noise, we could further increase the amplification without the saturating the output voltage.

**Figure 12.**
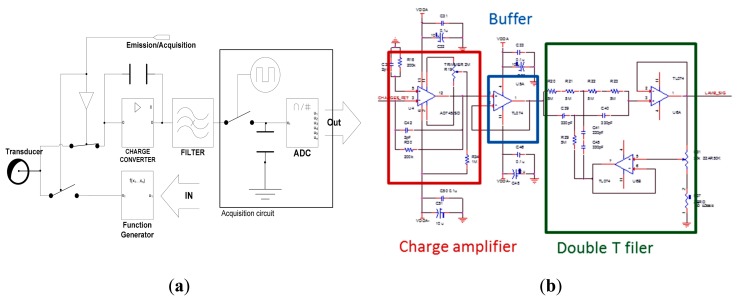

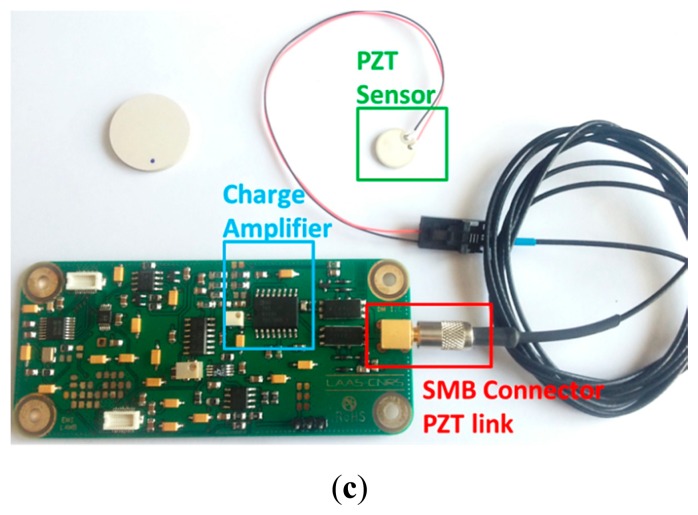
Smart sensor: (**a**) synopsis of the developed electronics; (**b**) analog interfacing circuit; (**c**) photo of the developed electronics.

Finally, using the previous circuits, we built a smart node (Figure 12), based on the charge amplifier presented in Figure 7b, a 50 Hz notch filter and an external mixed analog/digital instrumentation electronics to demontrate the faisabilité of the concept.

## 4. Damage Detection into Real Aircrafts Specimens Using the Developed Sensor

### 4.1. Delamination Detection into Composite Material Using Guided Waves

Under a harmonic electrical excitation, the PZT patch creates a periodical radial strain that is transferred to the structure through the interface layer by a periodical shear stress:

(14) $$T\left[x,t\right]=T_{\text{interface}}\;\left(x\right)e^{i\omega t}$$

The induced structure displacement can be decomposed into two uncoupled differential equation parts using the Helmholtz decomposition [15]:

(15) $$\frac{\partial^{2}\phi }{\partial {\text{x}}^{2}}+\frac{\partial^{2}\phi }{\partial {\text{y}}^{2}}+\frac{\Omega^{2}}{{\text{c}}_{\text{p}}^{2}}\Phi =0$$

(16) $$\frac{\partial^{2}\psi }{\partial {\text{x}}^{2}}+\frac{\partial^{2}\psi }{\partial {\text{y}}^{2}}+\frac{\Omega^{2}}{{\text{c}}_{\text{s}}^{2}}\psi =0$$

**Figure 13.**
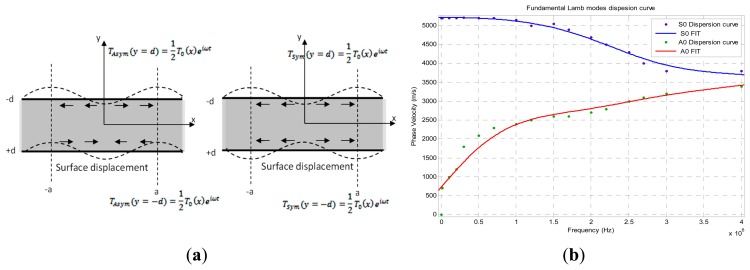
(**a**) Typical structure of fundamental symmetric and anti-symmetric Lamb wave modes respectively S_0_ and A_0_; (**b**) Fundamental Lamb modes S_0_, A_0_ dispersion curves.

Equation (15) governs the longitudinal wave modes and Equation (16) governs the transverse wave modes (Figure 13a). ${\text{c}}_{\text{p}}^{2}=(\lambda +2µ)/\rho$ and ${\text{c}}_{\text{s}}^{2}=µ/\rho$ are the longitudinal and the transverse, shear velocity, respectively.

For isotropic or quasi-isotropic structures the resolution of Equations (15) and (16) yields the characteristic equation of Lamb wave also called the Rayleigh-Lamb [16] equation:

(17) $$\frac{\tan\;pd}{\tan\;qd}=-{\left[\frac{4k^{2}pq}{(k^{2}-{q2)}^{2}}\right]}^{\pm 1}$$

where +1 corresponds to a symmetrical mode of propagation and −1 to the asymmetric mode (Figure 13); $p^{2}=\omega^{2}/c_{p}^{2}-k^{2}$ and $q^{2}=\omega^{2}/c_{s}^{2}-k^{2};$ *k* is the wave number.

Equation (17), allows for a fixed operating frequency excitation and for a set host structure thickness the identification of generated Lamb waves modes. Therefore, it can predict their dispersion phase velocity as shown in Figure 13b.

Due to the complex nature of aircraft structures, multiple echoes and wave interference are produced in the host structure [17]. The ribs and stiffeners make the study of Lamb wave propagation into the structure very complex and therefore make the damage diagnosis using conventional techniques based on predictive behaviors unsuitable [18]. One of the solution that we developed, consist of a smart minimization of Lamb modes number using Equation (17) and the dispersion curves. Actually, the number of generated modes is closely linked to the excitation frequency [19]. Therefore, in order to minimize the complexity of the acquired signal we should fix an operating point that ensures the best reduction of coexisting generated Lamb modes [18].

**Figure 14.**
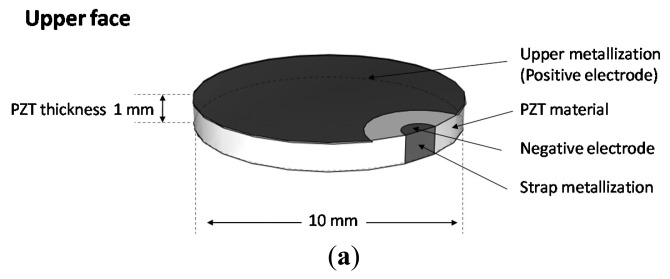

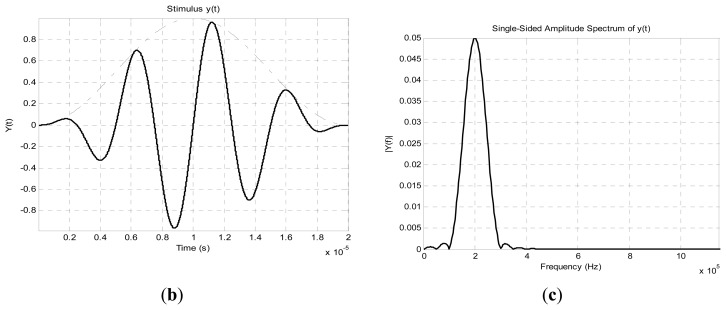
(**a**) Designed sensor; (**b**) Generated stimulus in time domain: Pure sinus modulated using a Hanning window; (**c**) Generated stimulus in frequency domain.

The spectral response of the excitation signal should be very narrow and focused around the chosen operating frequency. To achieve this aim, we used a Hanning windowed sinus waveform of 200 kHz (Figure 14b,c) which ensures the generations of two modes A_0_ and S_0_ for AL2024 structure thicknesses ranging from 1 to 4 mm [18–20]. The sensor used has a diameter of 9.5 mm and is 1 mm thick (Figure 14a). The equation of the used waveform is:

(18) $$x\left(t\right)=\frac{1}{2}\left[1-\cos\;\left(\frac{2\pi t}{T_{H}}\right)\right]\;\sin\;\left(2\pi \omega t\right)$$

with:

$$T_{H}=\frac{N_{B}}{f}\;\text{And}\;N_{B}=4$$

### 4.2. Experimental Results

**Figure 15.**
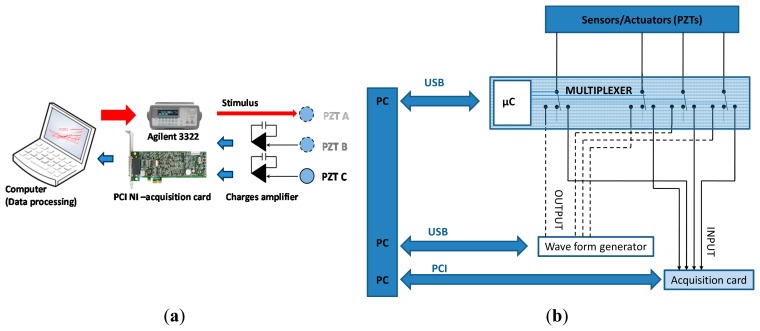

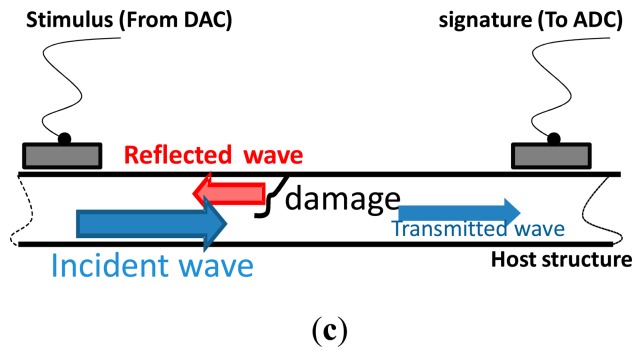
(**a**) Schematic of the experimental setup: The stimulus is generated by PZT A-1 and received by PZT B-2 and C-3. The data is recorded using a NI acquisition card at a rate of 2.5 Msamples/s; (**b**) Detailed synoptic of the experimental setup; (**c**) Pitch catch principle.

**Figure 16.**
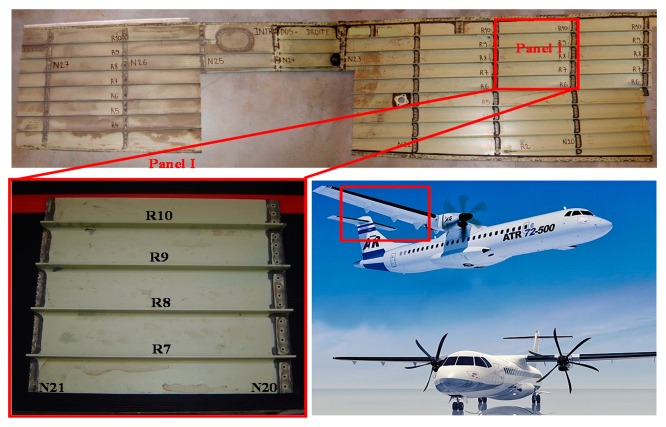
Extracted composite specimen from an ATR72 wing pannel.

**Figure 17.**
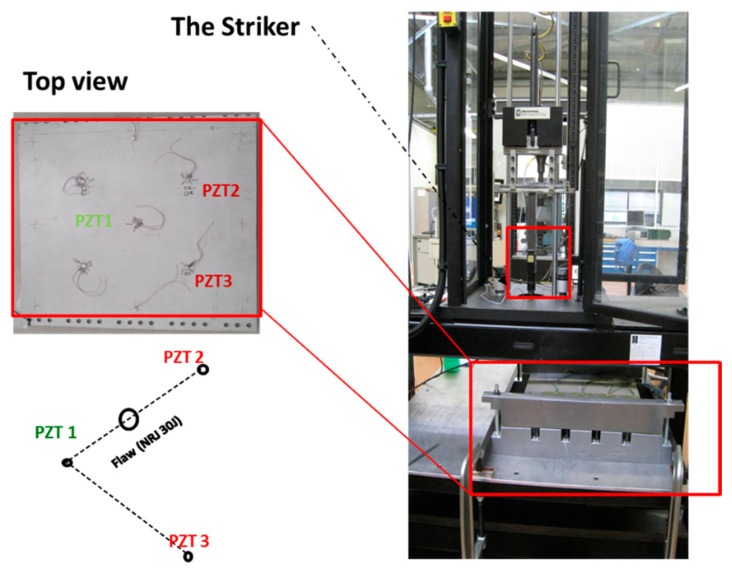
Experimental setup: The stimulus is generated by the PZT 1 and received by PZT 2 and 3.

To demonstrate the feasibility of damage detection in aircraft structure specimens, we installed a specific test bench (Figure 15a,b), based on an Agilent 32200 A waveform generator and a NI-PCI acquisition card. The first instrument is used to generate a specific stimulus which is presented in Figure 15. On the other hand, the acquisition card is used to capture the global structure response through the sensor' generated signal. The used test structure was extracted from the right wing of an ATR-72 (Figure 16) [21]. It measures 49.5 × 46 cm and is made from composite stratified carbon fibers. Three PZT sensors were pasted onto the surface of the structure. A delaminating impact of 30 J was applied between PZT1 and PZT2 using a calibrated impact machine (Figure 17). At this impact energy, the delaminating zone presents no visible external damage.

To ensure a correct digitalization, the PCI acquisition card was configured to reach a sampling frequency of 2.5 Msamples/s. For the purpose of this article, we limited the saved data to 600 samples. The signals analysis was performed using wavelet decomposition [8–22]. In fact compared to the other joined time frequency methods, the wavelets transform represents the best compromise between the time and the frequency resolution [23]. Actually, due to the special proprieties of the used wavelet, the resolution in time is much higher at high frequencies. This specificity makes wavelets analysis mostly used to localize the exact time of a specific and time narrow event.

**Figure 18.**
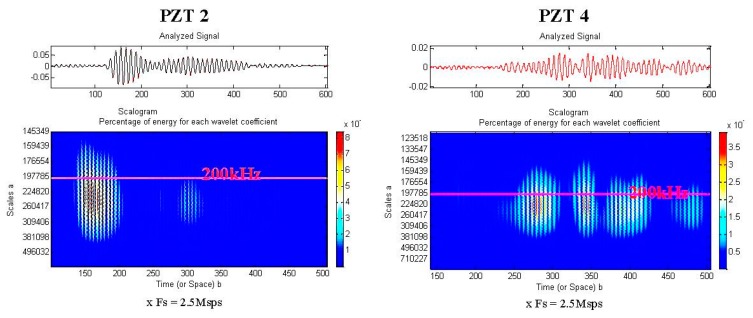
Time-frequency analysis: Notice the difference between the signature of PZT 4 and PZT 2. The delamination area seems to act as a passive filter.

As depicted in Figure 18, the response of the damaged area is clearly different from the response of the healthy zone. Finally, using a simple visual comparison between acquired signatures, we were capable to detect mechanical flaws caused by delaminating impacts. In fact the explanation of the phenomenon is directly linked to the alterations that damage induces to the incident Lamb wave during its travel into the structure. A portion of the energy is reflected as presented in Figure 15c while the rest is transmitted.

## 5. Conclusions/Outlook

Ground health monitoring using Lamb waves is a powerful method capable of detecting delamination flaws in complex composite stratified structures. Based on piezoelectric material, the required sensors are relatively cheap and thus suitable for industrial applications such as aerospace structural health monitoring. Reconfigurable electronics based on a switched capacitor is currently under development by the authors to demonstrate the strength of guided waves in different structural configurations. In addition, more studies should be done to investigate the influence of environmental parameters, such as temperature and humidity, on the acquired baselines. New algorithms based on simpler calculations are to be studied for real time detection and embedded processing.

## Figures and Tables

**Table 1.** (**a**) Properties of different piezoelectric materials; (**b**) Properties of different PZT types.

**Property**	**Unit**	**Film PVDF**	**PZT (PbZrTiO_3_)**	**BaTiO_3_**
Density	kg/m^3^	1780	7500	5700
Relative permittivity	ε/ε_0_	12	1200	1700
d_31_	10^−12^ C/N	23	110	78
g_31_	10^−3^ Vm/N	216	10	5
k_31_	at 1 kHz	0.12	0.30	0.21
E	GPa	3	60	110
Acoustic Z	10^6^ kg/m^2^s	2.7	30	30

**(a)**				

**Navy Type**	**PZT-5A**	**PZT-8**	**PZT-5J**	**PZT-5H**

Property	II	III	V	VI
Permittivity	1750	1100	3000	3200
k_33_	0.72	0.64	0.73	0.74
k_T_	0.5	0.46	0.54	0.54
k_31_	0.61	0.51	0.63	0.65
d_33_ (pC/N)	390	300	580	650
|d_31_| (pC/N)	175	100	220	250
g_33_ (mV·m/N)	40	29	21	23
|g_31_| (mV·m/N)	12	10	9	9

**(b)**				
